# Aberrant DNA methylation patterns in diabetic nephropathy

**DOI:** 10.1186/2251-6581-13-69

**Published:** 2014-06-24

**Authors:** Zhila Maghbooli, Bagher Larijani, Solaleh Emamgholipour, Manochehr Amini, Abbasali Keshtkar, Parvin Pasalar

**Affiliations:** 1 Endocrinology and Metabolism Clinical Sciences Institute of Tehran University of medical sciences, EMRI, 5th floor, Shariati Hospital, North Karegar Avenue, P.O Box: 1411413137, Tehran, Iran; 2 Clinical Biochemistry Department, School of Medicine, Faculty of Medicine Tehran University of medical sciences, EMRI, 5th floor, Shariati Hospital, North Karegar Avenue, P.O Box: 1411413137, Tehran, Iran; 3 Nephrology Department, Shariati Hospital, Tehran University of Medical Sciences, EMRI, 5th floor, Shariati Hospital, North Karegar Avenue, P.O Box: 1411413137, Tehran, Iran

**Keywords:** Type 2 diabetes, Diabetic nephropathy, Albuminuria, Global DNA methylation, Epigenetic

## Abstract

**Background:**

The aim of this study was to evaluate whether global levels of DNA methylation status were associated with albuminuria and progression of diabetic nephropathy in a case-control study of 123 patients with type 2 diabetes- 53 patients with albuminuria and 70 patients without albuminuria.

**Methods:**

The 5-methyl cytosine content was assessed by reverse phase high pressure liquid chromatography (RP-HPLC) of peripheral blood mononuclear cells to determine individual global DNA methylation status in two groups.

**Results:**

Global DNA methylation levels were significantly higher in patients with albuminuria compared with those in normal range of albuminuria (p = 0.01). There were significant differences in global levels of DNA methylation in relation to albuminuria (p = 0.028) and an interesting pattern of increasing global levels of DNA methylation in terms of albuminuria severity.

In patients with micro- and macro albuminuria, we found no significant correlations between global DNA methylation levels and duration of diabetes (p > 0.05). In both sub groups, there were not significant differences between global DNA methylation levels with good and poor glycaemic control (p > 0.05). In addition, in patients with albuminuria, no differences in DNA methylation levels were observed between patients with and without other risk factors including age, gender, hypertension, dyslipidaemia and obesity.

**Conclusions:**

These data may be helpful in further studies to develop novel biomarkers and new strategies for clinical care of patients at risk of diabetic nephropathy.

## Background

Diabetic nephropathy (DN) is one of the most serious micro vascular complications and remains a leading cause of end-stage renal disease (ESRD) and kidney replacement therapy [1]. Consequenctly, DN is associated with reduced life expectancy and increased morbidity, thereby imposing a significant burden on national healthcare system in diabetic care [2,3].

The persistent albuminuria has been considered to be a central hallmark of DN and also a clinically informative predictor of kidney damage in diabetes patients [4,5]. Albuminuria was categorized into two types: to micro albuminuria and macro albuminuria, and clinically, micro albuminuria is used extensively as a helpful predictor of first stage of kidney damage. However, despite its capacity to herald kidney damages for clinicians, micro albuminuria is not a specific biomarker for prediction and prevention of DN prior to the onset of this devastating complication. In principle, there are numerous cellular and molecular defects prior to appearing a clinical symptom and researchers still represent a major medical challenge in the hope to unravel an easy and accurate way to detect DN before its beginning. Moreover, better understanding of the disease pathogenesis might be helpful to identify new biomarkers and also novel mechanisms underlying DN.

Although DN is a common disorder with a heritable manner affected by genetic factors [6-8], this heritability could not be fully explained by strict genetic inheritance patterns [9]. It is now accepted that the initiation and progression of DN are a result of complex interactions between genetic and environmental factors that could be explained by epigenetic modifications [10,11]. Environmental signals could alter the intracellular pathways by chromatin modifiers and regulate gene expression patterns leading to diabetes and its complications. A hyperglycaemic environment in diabetic patients causes an imbalance in metabolic homeostasis that may alter gene expression [12]. Recent epigenetic studies have focused on how gene transcription controls cell behaviour in response to environmental changes [13]. It will help to recognize novel approaches to inhibit, attenuate, or reverse the persistent deleterious consequences of diabetes in the kidney.

DNA methylation of cytosine residues is a heritable epigenetic modification of chromatin structure [14]. Alterations in genomic methylation patterns have been described in several complex disorders such as cancers that making it interesting for researchers as a plausible biomarker and a target of treatment. Emerging evidence regarding the relationship between a hyperglycaemic environment and alterations in DNA methylation, suggests that evaluation of global DNA methylation could be used as a possible biomarker to identify diabetic complications [13]. Thus far, there are few studies investigating DNA methylation alterations in DN and chronic kidney disease (CKD) patients [15-17].

They have demonstrated the role of DNA methylation patterns in DN and also methylation alterations in key genes to be responsible for DN development. However, the contribution of global DNA methylation associated with albuminuria and DN remains to be explored. Here, we evaluated whether global levels of DNA methylation status are associated with albuminuria and progression of DN.

## Material and methods

### Study population

This clinical-based case–control study of diabetes patients was conducted from July 2102 to September 2013 in a referral diabetes clinic that is affiliated with the Tehran University of Medical Sciences. The study was approved by the Ethics Committee of the Endocrinology and Metabolism Research Institute and written informed consent was obtained from all participants.

The case subjects were type 2 diabetic patients with persistence micro- and macro albuminuria (n = 53). The control subjects (n = 70) were diabetic patients with urinary albumin excretion values within the normal range, without retinopathy and history of hypertension and not on antihypertensive medications.

The primary exclusion criteria for case and control groups were a history of cancer, chronic disorders such rheumatoid arthritis, liver disease, clinical sings of acute infection, current smoker, pregnancy at the time of study and type 1 diabetes.

The diagnosis of type 2 diabetes was carried out and/or confirmed following American Diabetes Association criteria; which include a fasting blood glucose ≥ 126 mg/dL on two separate occasions, random (non-fasting) blood glucose ≥ 200 mg/dL on two separate occasions, or a blood glucose > 200 mg/dL at 2 hours during a standard oral glucose tolerance test.

To investigate micro- and macro albuminuria, a urine albumin-to-creatinine ratio was determined from a random urine collection of all patients and classified as normal (urine microalbumin: creatinine ratio ≤ 30 μg/mg), micro albuminuria (urine microalbumin: creatinine ratio >30 μg/mg and ≤ 299 μg/mg) and macro albuminuria (urine microalbumin: creatinine ratio ≥ 300 μg/mg) at least on two separate occasions.

### Data collection and measurements

At first visit, demographic characteristics and clinical features including sex, age, age at diabetes diagnosis, diabetes duration, cigarette smoking status, and current use of medications were evaluated with a questionnaire. Standard anthropometric techniques were used to measure height, and weight. Body mass index was calculated as body weight (kg)/(height (m)^2^. Blood pressure was measured twice after participants were seated for at least for 10 minutes. Blood samples were taken after an overnight fast of 10-14 hours. The separated sera was kept at -80°C until analysis. Serum levels of glucose, total cholesterol, high-density lipoprotein (HDL), low-density lipoprotein (LDL), triglycerides (TG), blood urea nitrogen (BUN), uric acid, and creatinine were measured by enzymatic colorimetric assay [Pars-Asmun kits, Iran] using an auto analyzer [Hitachi 902, Japan]. The fasting serum insulin levels were assessed by an immunoenzymometric assay [Monobind Inc., USA]. The intra- and inter-assay coefficients of variation (CVs) for insulin were 5.9% and 9.2%, respectively. Glycated hemoglobin (HbA1c) levels were measured using ion exchange chromatography with a DS5 set [DREW, United Kingdom].

A random urine sample was collected the same day. Urine microalbumin and creatinine levels were measured by enzymatic colorimetric assay [Pars-Asmun kits, Iran] using an auto analyzer [Hitachi 902, Japan]. In case group urine microalbumin: creatinine ratio was >30 μg/mg (without any likely cause for error). For confirmation, a second urine sample was collected by the investigator at the next outpatient visit. The second urinalysis was handled the same way as the urinalysis for the first sample. If the second urine microalbumin: creatinine ratio was >30 μg/mg without cause for error, then the patients were eligible for the study as a case group.

### Definition of diabetes risk factors

Diabetes predisposing factors were defined based on ADA criteria [18]. Hypertension was defined in subjects with a BP ≥ 140/90 mm Hg or current use of high blood pressure medications. Dyslipidemia was defined as TG > 250 mg/dL and/or HDL < 35 mg/dL or using lipid-lowering medications. Glycemic control was categorized into poor and good glycemic control based on HbA1c ≥ 7% or HbA1c < 7%, respectively [18,19]. Obesity was classified based on BMI > 30 kg/m^2^.

### Genomic DNA preparation and DNA hydrolysis

Genomic DNA was extracted from peripheral blood mononuclear cells (PBMCs) using a phenol chloroform method. Genomic DNA hydrolysis was performed as described elsewhere [20]. Briefly, RNA was first removed by treating 50 μg DNA in 300 μL 1X Tris-EDTA buffer with RNase A and RNase T1 [both from Fermentas Life Science, Lithuania] at final concentrations of 100 μg/mL and 2000 units/mL, respectively, for 2 h at 37°C, which was followed by ethanol precipitation. The dissolved DNA was then digested with 50 μg/mL DNase I [Fermentas Life Science, Lithuania] for 14 h at 37°C, denatured by heating at 100°C for 3 minutes, and rapidly cooled on ice. Then, 2 volumes 30 mM sodium acetate pH 5.2 [Carlo Erba Reagenti SpA, Rodano, Italy] with ZnSO_4_ and nuclease P1 [Sigma-Aldrich, St. Louis, MO, USA] at final concentrations of 1 mM and 50 μg/ml, respectively, were added and the mixture incubated for a further 16 h at 37°C. Hydrolyzed DNA from all subjects was maintained at -80°C until analysis.

### Reverse phase high pressure liquid chromatography (RP-HPLC) analysis

#### Chemicals

Deionized water and HPLC-grade methanol [Merck, Darmstadt, Germany] were used in all experiments and all chemicals were of analytical reagent grade. The deoxynucleoside standards, 2-deoxycytidine 5-monophosphate sodium salt hydrate (dC), 2-deoxyguanosine 5-monophosphate sodium salt hydrate (dG), 2-deoxyadenosine 5-monophosphate (dA), thymidine 5-monophosphate disodium salt hydrate (dT), 5-methylcytidine (^met^C), were obtained from Sigma [St. Louis, MO, USA].

#### Instrumentation

A Waters 2487 dual absorbance detector chromatograph equipped with a Waters 1500 series HPLC pump was used in this study. The chromatographic column was a C8, 4.6 × 150 mm, 5 μm [Waters Spherisorb, Ireland].

### Analytical procedure

Isocratic RP-HPLC analysis was performed at 10°C with a flow rate of 0.6 ml/min. The separation was achieved with 50 mM ammonium phosphate dibasic (titrated to pH 4.0 with phosphoric acid) [Merck KGaA, Darmstadt, Germany] as the mobile phase. A detection wavelength of 280 nm was used to identify specific spectra peaks originating from nucleosides. The injection volume of all samples was 100 μL. The area under the peaks was used for quantitative analysis. For linear calibration curve was obtained of ten concentration solutions range of 0-10 μM/ml for dA, dG, dC and dT and range 0-0.34 μM/ml for ^met^C. The correlation coefficient of the curve was 0.999 for every compound. All samples were measured in duplicate. Coefficient of variation of deoxinoclotides between experiments ranged from 1.25% to 2.3%. The 5methyl-cytosine percentage was calculated using the equation: 5*Methyl* - *cyto* sin *e*(%) = 5*Methyl* - *cyto* sin *e*/(5*Methyl* - *cyto* sin *e* + *Cyto* sin *e*) ∗ 100.

### Data analysis

For the analysis, we used the first urine collection from patients with normal range of albuminuria (control group) and the second confirmatory urine collection with >30 μg/mg for patients with persistent microalbuminuria (case group).

Statistical analysis was conducted using SPSS software (version 16). As certain data, including fasting serum LDL, TG, and insulin levels did not have normal distributions; log transformation was applied to correct their normality distribution. Student’s *t*-test was used to compare the differences of global DNA methylation levels in diabetic patients with and without albuminuria. One-way ONOVA test was used to compare the clinical and laboratory features in subgroups; normal, micro- and macro albuminuria. Kruskal Wallis test was used for variables not normally distributed. A Pearson correlation was applied to examine correlation of clinical, laboratory findings and diabetes risk factors with global DNA methylation levels. Numerical variables are reported as the mean ± standard error and categorical variables are presented as percentages. Two-tail p-values less than 0.05 were considered as statistically significant.

## Results

### Clinical characteristic and laboratory features

The clinical characteristics of the study population in each subgroup are shown in Table 1. Patients with micro and macro albuminuria were older, and had longer duration of diabetes, higher HbA1c, TG, BUN and uric acid and lower HDL serum levels.

**Table 1:** The clinical characteristics in patients with micro- and macro albuminuria (case group) and control group

**DN risk factors**	**Patients without diabetic nephropathy (n = 70)**	**Patients with micro-albuminuria (n = 35)**	**Patients with macro-albuminuria (n = 18)**	**p-value**
Age-years	55.41 ± 0 .79	58.30 ± 0.99	58.23 ± 1.59	0.057
Sex-men (%)	25 (35.7)	19 (66.7)	12 (66.7)	0.030
Duration of diabetes-years	9.58 ± 0.67	14.34 ± 1.17	15.27 ± 1.27	0.0001
Age of onset-years	45.34 ± 0.94	44.00 ± 1.39	43.94 ± 1.99	0.650
Glycated haemoglobin-%	7.32 ± 0.17	8.25 ± 0.35	8.82 ± 0.54	0.002
Total cholesterol-mg/dl	153.12 ± 4.79	156.00 ± 6.56	170.05 ± 13.52	0.327
LDL-mg/dl	77.62 ± 1.02	81.28 ± 1.04	87.09 ± 1.07	0.342
HDL-mg/dl	50.22 ± 1.32	43.37 ± 1.69	43.00 ± 2.65	0.003
TG-mg/dl	109.64 ± 1.07	144.54 ± 1.07	162.18 ± 1.14	0.009
BMI-Kg/m^2^	27.90 ± 0.52	30.78 ± 1.01	28.66 ± 1.15	0.021
FBS-mg/dl	142.65 ± 6.01	154.65 ± 11.15	157 ± 14.13	0.467
insulin-μUI/l	9.55 ± 1.07	13.18 ± 1.20	11.48 ± 1.23	0.215
BUN-mg/dl	16.49 ± 1.00	24.52 ± 2.76	30.13 ± 2.38	0.0001*
Uric acid-mg/dl	4.95 ± 0.16	5.31 ± 0.26	10.67 ± 4.14	0.0001*

*P-value for kruskal Wallis test.

A family history of diabetes in primary relatives was reported for 77.4% in patients with albuminuria (combined patients with micro- and macro albuminuria) and 60% of patients without albuminuria (with normal range of albuminuria), respectively (p = 0.04). The prevalence of obesity in patients with albuminuria (51%) was higher than those with normal albuminuria (30%) (p = 0.019). Poor control of glycemia was observed on 71.7% of patients with albuminuria compared with 54.3% of patients with normal albuminuria (p = 0.049). There was no significant difference in the prevalence of dyslipidaemia in patients with and without albuminuria, 79.2% and 84.3%; respectively (p = 0.47). The prevalence of coronary artery disease was observed in 17% of patients with albuminuria and 10% in patients without albuminuria.

### Global DNA methylation and albuminuria

**Figure 1:**
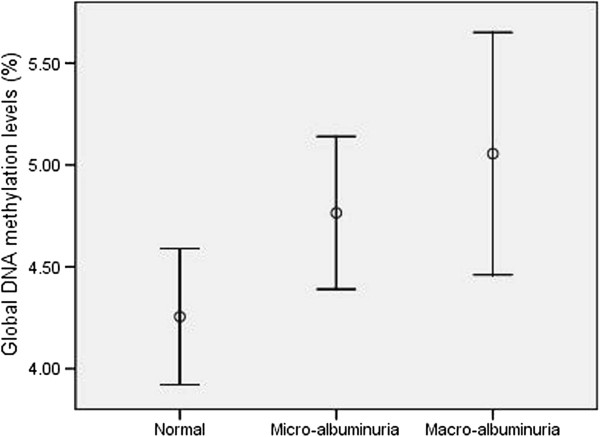
**Global DNA methylation levels related to severity of albuminur.** Error bares represent 95% CI for mean levels of global DNA methylation in patients with normal range of albuminuria, micro- and macro albuminuria.

Global DNA methylation levels were significantly higher in patients with albuminuria compared with those without albuminuria (4.86 ± 0.15 vs. 4.25 ± 0.16, respectively, p = 0.01). There was significant increasing trend in global DNA methylation levels in relation to albuminuria (normal range of albuminuria: 4.25 ± 0.16, micro albuminuria: 4.76 ± 0.18, macro albuminuria: 5.05 ± 0.28 (F = 3.75, p = 0.028) (Figure 1).

### Quantification of global DNA related to age and gender

No significance differences were observed in global DNA methylation levels between men and women in three groups (p > 0.05). There was also no significant correlation between age and DNA methylation levels in three groups (p >0.05). Patients with albuminuria (combined patients with micro- and macro albuminuria) had higher levels of DNA methylation independent of age and sex (p = 0.028).

### Global DNA methylation and glycemic control and duration of diabetes

In diabetic patients without albuminuria, there was significant correlation between global DNA methylation levels and duration of diabetes (p = 0.002). In contrast, there was no significant correlation between global levels of DNA methylation and duration of diabetes in patients with albuminuria (p = 0.84).

In subgroups patients with micro- and macro albuminuria, we found no significant correlation between duration of diabetes and global DNA methylation levels (Table 2).

**Table 2:** Correlation between Global DNA methylation levels and clinical and laboratory characteristic

**DN risk factors**	**Patients without diabetic nephropathy (n = 70)**		**Patients with micro-albuminuria (n = 35)**		**Patients with macro-albuminuria (n = 18)**	
	**R**	**P-value**	**R**	**P-value**	**R**	**p-value**
Age-years	0.07	0.55	0.18	0.31	-0.11	0.81
Sex-men	0.08	0.50	0.24	0.16	0.18	0.46
Duration of diabetes-years	0.37	0.002	0.12	0.50	0.06	0.81
Age of onset-years	-0.16	0.18	0.02	0.87	0.05	0.82
Glycated haemoglobin-%	0.06	0.59	0.02	0.91	0.42	0.08
Total cholesterol-mg/dl	-0.08	0.49	0.25	0.15	0.21	0.39
LDL-mg/dl	-0.05	0.66	0.20	0.25	0.26	0.29
HDL-mg/dl	0.02	0.85	-0.02	0.92	0.30	0.22
TG-mg/dl	-0.06	0.59	0.22	0.21	-0.21	0.39
BMI-Kg/m^2^	0.09	0.46	-0.31	0.08	-0.14	0.17
Insulin-μUI/l	0.22	0.07	-0.07	0.68	-0.25	0.32
FBS-mg/dl	0.07	0.57	-0.29	0.09	0.33	0.17
BUN-mg/dl	-0.22	0.07	-0.02	0.92	-0.38	0.11
Uric acid-mg/dl	0.001	0.99	0.19	0.28	-0.11	0.67

### Global DNA methylation and diabetic risk factors in patients with albuminuria

In case group; global DNA methylation levels were higher in patients with family history of diabetes in first-degree relatives than those without family history of diabetes but it was not significant (4.94 ± 0.17 vs. 4.61 ± 0.34, p = 0.37). There were no significant differences in global DNA methylation levels in patients with and without hypertension (p = 0.53), dyslipidaemia (p = 0.37), and obesity (p = 0.41).

## Conclusions

The main focus of current study was to examine global DNA methylation levels in relation to albuminuria in type 2 diabetic patients. We found higher levels of global DNA methylation in patients with micro- and macro albuminuria compared those with normal ranges of albuminuria even after adjusting age and sex. Interestingly, we did observe an interesting pattern of increasing global levels of DNA methylation in terms of albuminuria severity.

Although the role of DNA methylation in diabetic kidney gene expression remains poorly characterized, few studies have shown that altered DNA methylation at CpG sites to be associated with diabetic nephropathy [16,21]. Moreover, DNA methylation changes in key genes were correlated with the development of diabetic nephropathy [16,21]. Experimental studies and animal models have confirmed that long-lasting hyperglycaemia exposure causes DNA methylation changes in the promoter region of genes that are key components in diabetes and its complications [22-26].

This increasing body of evidence implicates that aberrant DNA methylation in response to hyperglycaemia may account for diabetic complications. It was revealed that the duration of diabetes prior to the onset of nephropathy was correlated with CpG methylation at those key genes [21]. Interestingly, our results showed glycaemic control and duration of diabetes were not associated with global DNA methylation in patients with albuminuria. It is accepted that chronic hyperglycaemia is the hallmark of development and progression of diabetic complications. This inconsistency may be due to the role of early hyperglycaemia exposure and may prime future transcriptional and metabolic profiles related to diabetes clinical endpoints [27-30]. There is also an increasing body of evidence to suggest that memory of early hyperglycaemic exposure can be retained in target cells that could subsequently alter gene expression patters even under euglycaemic conditions [27,31-35]. As broadly shown in epidemiological studies and trials, good glycaemic control could not prevent diabetes progression and associated vascular complications in most diabetic patients and early prevention of hyperglycaemia is more important in prevention of diabetes complications [36-39]. Our results, which are consistent with these studies, indicate that aberrant DNA methylation alterations might occur in response to a chronic hyperglycaemic environment rather than glucose fluctuations that arisen by glycaemic control. Moreover, the main known risk factors that attend to diabetic nephropathy such as dyslipidaemia, hypertension, inflammation and oxidative stress could alter DNA methylation patterns [40-43].

In the current study, we observed no association between global DNA methylation levels and obesity, dyslipidemia and hypertension in patients with albuminuria. In contrast, higher methylation levels in peripheral blood cell genomes were reported in essential hypertensive patients compared with subjects without hypertension [41]. Also, the importance of DNA methylation modification to the development of cardiovascular disease is evidenced in some studies [44,45]. Additionally, in another study in chronic kidney disease patients, global levels of DNA hypermethylation were correlated with systemic inflammation [40].

In a study in non-diabetic patients, a positive correlation was observed between Long Interspersed Nucleotide Element 1 (LINE-1) DNA methylation (an index of global DNA methylation) and total cholesterol, triglycerides and LDL-cholesterol concentrations. A negative association was also observed between LINE-1 methylation and HDL cholesterol concentration [46]. While, in a study in obese patients, DNA methylation changes in peripheral blood leukocytes were assessed and showed no methylation differences in CpG sites between the obese and lean groups [47].

Although we detected no significant association between global DNA methylation levels and obesity, hypertension, and dyslipidemia in patients with albuminuria, a possible relationship between these factors and DNA methylation cannot be ignored. How these characters modulate intracellular pathways inducing epigenetic alterations and cause long-lasting effects is not completely understood.

Our study takes a global approach to determine DNA methylation alterations in diabetic patients related to albuminuria. The capacity to analyse DNA methylation of the entire genome could provide an overall overview of cellular DNA methylation levels and be also crucial for clear the mechanisms by which gene-environment interplay leads to altered DNA methylation [48]. It also will enable us to understand the role of DNA methylation in normal development as well as disease state. Since the design of present study is a clinical case-control, it could not show causality between global DNA methylation and severity of albuminuria. Therefore, future studies with longitudinal design will be required to confirm this possibility. In this study, we evaluated global DNA methylation using HPLC assay which would be expected to be a standard method with higher sensitivity and more reproducibility than other methods used to determine global DNA methylation levels [49]. HPLC is a quantitative method and the single nucleotides are separated according to size and both cytosine and methylated cytosine are quantified [50].

Our study measured global DNA methylation levels in PBMCs. It is expected that DNA methylation is a specific- tissue manner and global DNA methylation tissue patterns have been found to significantly differ between tissue types [51]. However, human tissues availability set limitations and are not always accessible to evaluate global DNA methylation changes. In addition, PBMCs have been used for exploring gene expression in various diseases and predicting clinical outcome [52-54]. PBMCs can also reflect the effect of metabolic and environmental factors on chromatin structure and DNA methylation [55] and can useful in understanding etiology of complex disorders modulated by gene– environment interplay such as diabetes.

In summary, we have shown that global DNA methylation alterations associated with albuminuria and there was a significant increasing trend in global DNA methylation levels in relation to severity of albuminuria. Advances in understanding of epigenetic modifications in the progression of diabetic nephropathy could be helpful to clear pathogenic pathways and subsequent translation of this information into development of novel biomarkers and new strategies for clinical care of patients at risk of diabetic nephropathy.

## Abbreviations

DN: Diabetic nephropathy; PBMC: peripheral blood mononuclear cells.

## Competing interests

The authors declare that they have no competing interests.

## Authors’ contributions

All authors designed the study. ZM and MA gathered the clinical data. ZM and SE performed experiments. ZM, BL and AK analyzed the data. ZM, BL, SE, MA, and AK wrote the main paper. All authors discussed the results and commented on the manuscript at all stages. All authors read and approved the final manuscript.
